# CRISPRdirect: software for designing CRISPR/Cas guide RNA with reduced off-target sites

**DOI:** 10.1093/bioinformatics/btu743

**Published:** 2014-12-09

**Authors:** Yuki Naito, Kimihiro Hino, Hidemasa Bono, Kumiko Ui-Tei

**Affiliations:** ^1^Database Center for Life Science (DBCLS), ^2^National Institute of Genetics, Research Organization of Information and Systems, 1111 Yata, Mishima, Shizuoka 411-8540, Japan and ^3^Department of Biological Sciences, Graduate School of Science, University of Tokyo, 7-3-1 Hongo, Bunkyo-ku, Tokyo 113-0033, Japan

## Abstract

**Summary:** CRISPRdirect is a simple and functional web server for selecting rational CRISPR/Cas targets from an input sequence. The CRISPR/Cas system is a promising technique for genome engineering which allows target-specific cleavage of genomic DNA guided by Cas9 nuclease in complex with a guide RNA (gRNA), that complementarily binds to a ∼20 nt targeted sequence. The target sequence requirements are twofold. First, the 5′-NGG protospacer adjacent motif (PAM) sequence must be located adjacent to the target sequence. Second, the target sequence should be specific within the entire genome in order to avoid off-target editing. CRISPRdirect enables users to easily select rational target sequences with minimized off-target sites by performing exhaustive searches against genomic sequences. The server currently incorporates the genomic sequences of human, mouse, rat, marmoset, pig, chicken, frog, zebrafish, *Ciona*, fruit fly, silkworm, *Caenorhabditis elegans*, *Arabidopsis*, rice, *Sorghum* and budding yeast.

**Availability:** Freely available at http://crispr.dbcls.jp/.

**Contact:** y-naito@dbcls.rois.ac.jp

**Supplementary information:** Supplementary data are available at *Bioinformatics* online.

## 1 Introduction

Genome engineering is a promising technique to manipulate endogenous chromosomal DNA in a site-specific manner. A novel system that employs the prokaryotic immune defense system based on the clustered regularly interspaced short palindromic repeats (CRISPR) and CRISPR-associated (Cas) protein has been reported as a prominent genome engineering approach (Cho *et al.*, 2013; Cong *et al.*, 2013; Jinek *et al.*, 2013; Mali *et al.*, 2013). Recent studies utilize the RNA-guided endonuclease Cas9 from *Streptococcus pyogenes* and a guide RNA (gRNA), which acts as a guide to define the target site to introduce DNA double-stranded break. A remarkable advantage of the CRISPR/Cas system is that the target DNA sequence is recognized by simple base-pairing complementarity by the gRNA. Thus, the CRISPR/Cas system can be programmed only by changing the gRNA sequence, and the synthesis of the gRNA for targeting a specific gene is easy at low cost. However, it should be a critical issue to avoid the cleavage of the unintended off-target genes, since double-stranded break results in stable and heritable modification of the genome.

In this study, we present CRISPRdirect (http://crispr.dbcls.jp/), which provides efficient selection of CRISPR/Cas target sites with reduced numbers of potential off-target candidates. CRISPRdirect investigates the entire genome for perfect matches with each candidate target sequence (20 mer) and their seed sequence (12 or 8 mer) flanking the PAM. Users can also browse the detailed list of potential off-target sites that have partial complementarity with the selected sequence. The server incorporates genomic sequences of human, mouse, rat, marmoset, pig, chicken, frog, zebrafish, *Ciona*, fruit fly, silkworm, *Caenorhabditis elegans*, *Arabidopsis*, rice, *Sorghum* and budding yeast. Currently, several web servers are available for designing CRISPR/Cas gRNAs (Supplementary Table S1). CRISPR Design (Hsu *et al.*, 2013; Ran *et al.*, 2013) performs gRNA selection from an input sequence up to 250 bp, and gRNAs are scored based on predicted off-target interactions. E-CRISP (Heigwer *et al.*, 2014) ranks gRNAs according to on-target specificity and number of off-targets. E-CRISP, ZiFiT (Sander *et al.*, 2010), Cas9 Design (Ma *et al.*, 2013) and CHOPCHOP (Montague *et al.*, 2014) utilize Bowtie (Langmead and Salzberg, 2012) to perform off-target searches allowing mismatches. On the other hand, DNA2.0 gRNA Design Tool (https://www.dna20.com/eCommerce/startCas9) searches for perfect matches with 12 nt seed to identify off-target sites. These servers except CRISPR Design and ZiFiT can process at least 10 kbp of input sequence. Web servers for checking off-target sites for given 20 nt sequences are also available, such as Cas-OFFinder (Bae *et al.*, 2014) and GGGenome (http://GGGenome.dbcls.jp/). These web servers are useful for designing gRNAs for a few input sequences, but processing large number of input sequences requires a laborious process. Even in such cases, CRISPRdirect returns the results quickly and provides a convenient interface for automated gRNA design as described in the Data export and API section, making it a powerful tool for using CRISPR/Cas system on a genome-wide scale.

## 2 Web server implementation

### 2.1 Overview

**Fig. 1.**
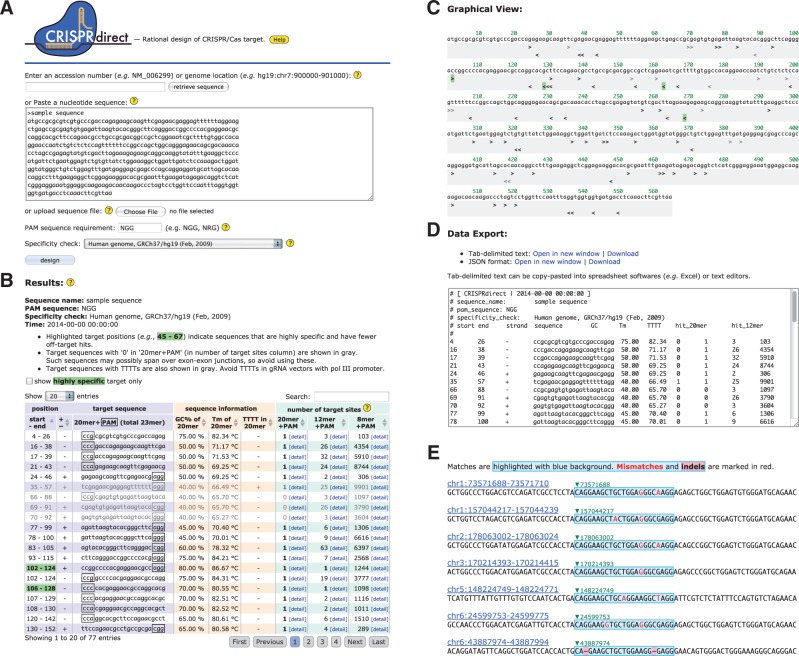
Screenshot from the CRISPRdirect web server. (**A**) Top page. The server accepts either an accession number or a nucleotide sequence as input. (**B**) Typical output of CRISPRdirect. A list of CRISPR/Cas target candidates is displayed. (**C**) A graphical view of target sites demonstrates the position and orientation of each site. (**D**) The results can be exported as tab-delimited text or in JSON format. (**E**) Detailed list of potential off-target sites which visualizes the positions of mismatches and gaps

The web server accepts an accession number, a genome coordinate or an arbitrary nucleotide sequence up to 10 kbp as input (Fig. 1A) and returns a list of CRISPR/Cas target candidates. Target sequences of 20 nt adjacent to the PAM sequence (e.g. NGG, NRG) are searched from both strands of the input sequence and listed as shown in Figure 1B. The list contains target position, target sequence, additional information on the sequence and the number of target sites in the genome. The additional information on the sequences such as GC content and calculated melting temperature (Tm) are provided, since previous report suggested that sgRNA sequences with very high or low GC content were less effective against their targets (Wang *et al.*, 2014). The presence or absence of TTTT (four consecutive T’s that cause pol III termination) in the target sequence is also indicated in order to avoid TTTT in gRNA vectors with pol III promoter. A detailed description of the web server is provided in Supplementary Methods.

### 2.2 Off-target evaluation

The number of target sites in the genome (Fig. 1B) is counted using Jellyfish (Marçais and Kingsford, 2011). The column ‘20 mer+PAM’ shows the number of hits with perfect matches for each target sequence (20 mer) adjacent to the PAM. Although the exact length of the completely complementary region necessary for cleavage by CRISPR nucleases is unknown, the mutations within the ‘seed’ sequence at 8–12 nt immediately adjacent to the PAM are known to impair cleavage, suggesting that this region is the most critical determinant of target specificity (Cong *et al.*, 2013; Fu *et al.*, 2013; Hsu *et al.*, 2013; Pattanayak *et al.*, 2013). Therefore, we built up the columns ‘12 mer+PAM’ and ‘8 mer+PAM’ in order to show the number of hits with perfect matches for their seed sequence (12 or 8 mer, respectively) adjacent to the PAM. Note that the numbers of hits displayed here include both on-target and off-target sites. For instance, one (‘1’) in these columns indicates that the sequence has only one perfect match with the intended target site. Any number greater than one indicates that there are some potential off-target sites. Thus, in terms of avoiding off-target editing, the smaller the number (but not zero) is, the better. Zero (‘0’) in these columns means that the sequence has no match in the genomic sequence; such sequences may possibly span over exon–exon junctions, so their use should be avoided. CRISPRdirect highlights the CRISPR/Cas targets that have relatively fewer off-target sites (Fig. 1B and C). A detailed list of off-target candidates can be investigated by clicking the ‘detail’ link (Fig. 1E). The searches allowing mismatches and gaps (insertions and/or deletions) are performed using GGGenome (http://GGGenome.dbcls.jp/) REST API developed by the authors’ group instead of widely used BLAST (Altschul *et al.*, 1990), because BLAST may overlook some potential off-targets as mentioned in our previous work describing siDirect (Naito *et al.*, 2004), a web server for designing functional siRNA with reduced off-target effects. GGGenome quickly searches short nucleotide sequences utilizing suffix arrays and inverse suffix links indexed on solid state drive (SSD). As shown in Supplementary Table S1, off-target searches allowing gaps are not yet available in other existing web tools. However, the most recent report shows that CRISPR/Cas9 system has off-target activity with insertions or deletions between target DNA and gRNA sequences (Lin *et al.*, 2014). Therefore, we consider that off-target searches allowing mismatches and gaps would be a more suitable procedure to list off-target candidates exhaustively. The positions of the mismatches and gaps are visualized in the list (Fig. 1E), which may help predict the potency of off-target editing.

CRISPRdirect incorporates genomic sequences of various organisms to perform off-target searches. Although *Xenopus laevis* has long been used as a preferred model organism among developmental biologists, we incorporated *X.tropicalis* genome instead of *X.laevis* genome, because *X.tropicalis* is diploid while *X.laevis* is allotetraploid which makes it difficult to select specific targets.

There are some loci that are difficult to select specific targets. Typical examples are the histone clusters (NM_021059, etc.) and ribosomal proteins (NM_022551, etc.), which are known to form multigene families. When designing CRISPR targets for such genes, users should manually investigate a detailed list of potential off-target sites (Fig. 1E) and select the sequence that has fewer off-target hits on unrelated loci. Alternatively, if site-specific gRNA could not be designed within intended region, multiple gRNA approaches would be considerable (Guilinger *et al.*, 2014; Ran *et al.*, 2013; Tsai *et al.*, 2014). For such strategy, graphical view of CRISPRdirect results which visualizes the position and orientation of target sites (Fig. 1C) would be helpful for selecting paired gRNAs.

### 2.3 Data export and API

The results can be exported as tab-delimited text or in JSON format from the bottom of the result page (Fig. 1D). Users can copy–paste the text results into a spreadsheet or text editor for downstream analysis. The results can also be downloaded as a separate file by clicking the ‘download’ link. Alternatively, tab-delimited text or JSON output can be obtained via API, which is convenient for users to design a number of CRISPR/Cas targets in an automated manner.

## Funding

Life Science Database Integration Project, National Bioscience Database Center (NBDC) of Japan Science and Technology Agency (JST) (to Y.N. and H.B.); Grant-in-Aid for Scientific Research from the Ministry of Education, Culture, Sports, Science and Technology (MEXT) of Japan (to Y.N. and K.U.-T.); Cell Innovation Program of MEXT (to K.U.-T.).

*Conflict of interest*: none declared.

## Supplementary Material

Supplementary Data
