# Supplementary material for: CRISPRdirect: software for designing CRISPR/Cas guide RNA with reduced off-target sites
Source: Bioinformatics. 2014 Dec 9;31(7):1120–3. doi: 10.1093/bioinformatics/btu743 (PMC4382898; doi:10.1093/bioinformatics/btu743)
Supplement: Supplementary Data [file supp_31_7_1120__index.html]

CRISPRdirect: software for designing CRISPR/Cas guide RNA with reduced off-target sites — CRISPRdirect: software for designing CRISPR/Cas guide RNA with reduced off-target sites — CRISPRdirect: software for designing CRISPR/Cas guide RNA with reduced off-target sites — Supplementary Data 

# CRISPRdirect: software for designing CRISPR/Cas guide RNA with reduced off-target sites

## Supplementary Data

files

**Files in this Data Supplement:**

- Supplementary Data - pdf file
- Supplementary Data - pdf file
