# Supplementary material for: CRISPRdirect: software for designing CRISPR/Cas guide RNA with reduced off-target sites
Source: Bioinformatics. 2014 Dec 9;31(7):1120–3. doi: 10.1093/bioinformatics/btu743 (PMC4382898; doi:10.1093/bioinformatics/btu743)
Supplement: Supplementary Data [file supp_btu743_CRISPRdirect_Methods.pdf]

Supplementary Information

CRISPRdirect: software for designing CRISPR/Cas guide RNA with reduced off-target sites

Yuki Naito, Kimihiro Hino, Hidemasa Bono and Kumiko Ui-Tei

Supplementary Methods

1 Input

CRISPRdirect accepts the following input:

- **Accession number** (e.g. NM_001187)
- **Genome coordinate** (e.g. hg19:chr7:900000-901000)
  These are converted into nucleotide sequence using TogoWS API (Katayama *et al.*, 2010) as follows:
  http://togows.org/entry/ncbi-nucleotide/NM_001187.fasta
  http://togows.org/api/ucsc/hg19/chr7:900000-901000.fasta
- **Nucleotide sequence** in FASTA or plain sequence up to 10 kbp
- **Text file upload**
  Plain text file (not MS Word .doc file) containing FASTA or plain nucleotide sequence.

2 Parameters

- **PAM sequence requirement** (default: NGG)
  Target sequences adjacent to the specified PAM sequence are searched using regular expression pattern matching. Currently an arbitrary 3 nt sequence using IUB codes (N, R, Y, ...) can be specified.

Code	Base	Comment
**R**	A, G	pu**R**ines
**Y**	C, T	p**Y**rimidines
**M**	A, C	a**M**ino
**K**	G, T	**K**eto
**S**	C, G	**S**trong
**W**	A, T	**W**eak
**H**	A, C, T	not G
**B**	C, G, T	not A
**V**	A, C, G	not T
**D**	A, G, T	not C
**N**	A, C, G, T	any

- **Specificity check database**
  The database used for checking on-target and off-target sites.

3 Databases for specificity checking

- **Human** (*Homo sapiens*) genome, GRCh37/hg19 (Feb, 2009) ftp://hgdownload.soe.ucsc.edu/goldenPath/hg19/bigZips/chromFa.tar.gz
- **Mouse** (*Mus musculus*) genome, GRCm38/mm10 (Dec, 2011) ftp://hgdownload.soe.ucsc.edu/goldenPath/mm10/bigZips/chromFa.tar.gz
- **Rat** (*Rattus norvegicus*) genome, RGSC 5.0/rn5 (Mar, 2012) ftp://hgdownload.soe.ucsc.edu/goldenPath/rn5/bigZips/chromFa.tar.gz
- **Marmoset** (*Callithrix jacchus*) genome, WUGSC 3.2/calJac3 (Mar, 2009) ftp://hgdownload.soe.ucsc.edu/goldenPath/calJac3/bigZips/calJac3.fa.gz
- **Pig** (*Sus scrofa*) genome, SGSC Sscrofa10.2/susScr3 (Aug, 2011) ftp://hgdownload.soe.ucsc.edu/goldenPath/susScr3/bigZips/susScr3.fa.gz
- **Chicken** (*Gallus gallus*) genome, ICGSC Gallus_gallus-4.0/galGal4 (Nov, 2011) ftp://hgdownload.soe.ucsc.edu/goldenPath/galGal4/bigZips/galGal4.fa.gz
- **Frog** (*Xenopus tropicalis*) genome, JGI 4.2/xenTro3 (Nov, 2009) ftp://hgdownload.soe.ucsc.edu/goldenPath/xenTro3/bigZips/xenTro3.fa.gz
- **Zebrafish** (*Danio rerio*) genome, Zv9/danRer7 (Jul, 2010) ftp://hgdownload.soe.ucsc.edu/goldenPath/danRer7/bigZips/danRer7.fa.gz
- **Sea squirt** (*Ciona intestinalis*) genome, JGI 2.1/ci2 (Mar, 2005) ftp://hgdownload.soe.ucsc.edu/goldenPath/ci2/bigZips/ScaffoldFa.zip
- **Fruit fly** (*Drosophila melanogaster*) genome, BDGP R5/dm3 (Apr, 2006) ftp://hgdownload.soe.ucsc.edu/goldenPath/dm3/bigZips/chromFa.tar.gz
- **Roundworm** (*Caenorhabditis elegans*) genome, WS220/ce10 (Oct, 2010) ftp://hgdownload.soe.ucsc.edu/goldenPath/ce10/bigZips/chromFa.tar.gz
- **Thale cress** (*Arabidopsis thaliana*) genome, TAIR10 (Nov, 2010) ftp://ftp.arabidopsis.org/home/tair/Sequences/whole_chromosomes/*.fas
- **Rice** (*Oryza sativa*) genome, Os-Nipponbare-Reference-IRGSP-1.0 (Oct, 2011) Genome assemblies (12 chromosomes) + Unanchored sequences from http://rapdb.dna.affrc.go.jp/download/irgsp1.html
- **Sorghum** (*Sorghum bicolor*) genome, Sorghum bicolor v2.1 (May, 2013) ftp://ftp.jgi-psf.org/pub/compgen/phytozome/v9.0/early_release/Sbicolor_v2.1/assembly/Sbicolor_v2.1_255.fa.gz

- **Silkworm** (*Bombyx mori*) genome, Bmor1 (Apr, 2008) ftp://ftp.ensemblgenomes.org/pub/metazoa/release-17/fasta/bombyx_mori/dna/Bombyx_mori.Bmor1.17.dna.toplevel.fa.gz
- **Budding yeast** (*Saccharomyces cerevisiae*) (S288C) genome, sacCer3 (Apr, 2011) ftp://hgdownload.soe.ucsc.edu/goldenPath/sacCer3/bigZips/chromFa.tar.gz

4 Counting the number of target sites

The number of target sites in the genome is counted using Jellyfish (Marçais and Kingsford, 2011), a fast, parallel *k*-mer counting tool for DNA sequences. Jellyfish is based on a multithreaded, lock-free hash table optimized for counting *k*-mers up to 31 nt. The hash tables for 23, 15, and 11-mers were created for counting the number of hits with perfect matches for each target sequence (20mer) or their seed sequence (12mer or 8mer) adjacent to the PAM (3mer), respectively. The hash tables were stored on solid state drive (SSD) in order to make lookup faster.

5 Detailed list of off-target sites

A detailed list of off-target candidates (Fig. 1E) are shown using GGGenome (http://GGGenome.dbcls.jp/) REST API:

http://GGGenome.dbcls.jp/*db*/*k*/*sequence*[.*format*]
db: genome database
k: maximum number of mismatches and gaps
sequence: nucleotide sequence
format: html, txt, json

GGGenome quickly searches short nucleotide sequences utilizing suffix arrays and inverse suffix links indexed on solid state drive (SSD). The positions of the mismatches and gaps are visualized so users can predict the potency of off-target editing.

6 API

CRISPRdirect results can be directly retrieved in HTML, tab-delimited text or JSON format by simply POSTing the following values to http://crispr.dbcls.jp/ using any kind of language or script.

- **userseq** (Required)
  A nucleotide sequence; FASTA format or a plain nucleotide sequence up to 10 kbp.
  sample value: ggctgccaagaacctgcaggaggcagaaga…
- **accession** (Optional)
  Retrieve sequence mode. Set an accession number to retrieve sequence from GenBank instead of designing CRISPR/Cas targets. Genome location is also accepted for hg19, mm10, rn5, galGal4, xenTro3, danRer7, ci2, dm3, ce10 and sacCer3. The variable userseq should be null. The variable format should be 'html' or 'txt'.
  sample value: NM_001187, hg19:chr7:900000-901000
- **pam** (Optional)
  An arbitrary 3 nt sequence using IUB codes can be specified.
  sample value: NGG (default), NAG, …
- **db** (Optional)
  Set species for off-target searching.
  sample value: hg19 (human; default), mm10 (mouse), ...
- **format** (Optional)
  Output format.
  sample value: html (default), txt, json
- **download** (Optional)
  Set 'download' to download result as a file.
  sample value: download

References

Freier,A.M. *et al.* (1986) Improved free-energy parameters for predictions of RNA duplex stability. *Proc. Natl Acad. Sci. USA*, **83**, 9373-9377.

Katayama,T. *et al.*, (2010) TogoWS: integrated SOAP and REST APIs for interoperable bioinformatics Web services. *Nucleic Acids Res.*, **38**, W706-W711.

Marçais,G. and Kingsford,C. (2011) A fast, lock-free approach for efficient parallel counting of occurrences of *k*-mers. *Bioinformatics*, **27**, 764-770.

Panjkovich,A. *et al.* (2005) Comparison of different melting temperature calculation methods for short DNA sequences. *Bioinformatics*, **21**, 711-722.
