# Supplementary material for: CRISPRdirect: software for designing CRISPR/Cas guide RNA with reduced off-target sites
Source: Bioinformatics. 2014 Dec 9;31(7):1120–3. doi: 10.1093/bioinformatics/btu743 (PMC4382898; doi:10.1093/bioinformatics/btu743)
Supplement: Supplementary Data [file supp_btu743_CRISPRdirect_TableS1.pdf]

Supplementary Table S1

Web server	Reference	Max input sequence length	Off-target search: Supported organisms	Perfect matches	Mismatches	Insertions and deletions	Ranking methods	API
**CRISPR Design** (http://crispr.mit.edu/)	Hsu *et al.* (2013) *Nat. Biotechnol.*, **31**, 827-832. Ran *et al.* (2013) *Cell*, **154**, 1380-1389.	250 bp	human, mouse, zebrafish, *C. elegans*, rat, fruit fly, rabbit, pig, possum, chicken, *Arabidopsis*, dog, mosquito, stickleback	yes	yes	no	Scores based on faithfulness of on-target activity computed as 100% minus a weighted sum of off-target hit-scores, which are computed by taking into account total number of mismatches, mismatch absolute position, and mean pairwise distance between mismatches.	no (batch mode available, but takes very long time for large amount of inputs)
**E-CRISP** (http://www.e-crisp.org/)	Heigwer *et al.* (2014) *Nat. Methods*, **11**, 122-123.	more than 10 kbp	fly, *Arabidopsis*, human, zebrafish, *C. elegans*, mouse, rat, budding yeast, frog, dog, purple false brome, rice, medaka, maize, smut fungi, *Toxoplasma*, three-spined stickleback, black cottonwood, pig	yes	yes	no (off-targets with gaps are not detected as described)	Computes Specificity score, Annotation score, and Efficiency score. Specificity score starts with 100, for every off-target subtract (20-%mismatches).	no (supports multi FASTA file upload)
**ZiFiT** (http://zifit.partners.org/)	Sander *et al.* (2010) *Nucleic Acids Res.*, **38**, W462-W468.	1000 bp	human, rat, mouse, zebrafish, fruit fly, *C. elegans*, mosquito, *E. coli*	yes	yes	no	–	no (supports multi FASTA input, but off-target searches are not performed)
**Cas9 Design** (http://cas9.cbi.pku.edu.cn/)	Ma *et al.* (2013) *Biomed. Res. Int.*, **2013**, 270805.	more than 10 kbp	human, mouse, rat, zebrafish, fruit fly, *Arabidopsis*, tomato, sheep, honeybee	yes	yes	no	Sorted by both number of mapping hits and number of overlapping SNPs. gRNAs with only 1 mapping and 0 SNP are highlighted in green and ordered on the top.	no
**CHOPCHOP** (https://chopchop.rc.fas.harvard.edu/)	Montague *et al.* (2014) *Nucleic Acids Res.*, **42**, W401-W407.	more than 10 kbp	mosquito, *Arabidopsis*, *C. elegans*, fruit fly, zebrafish, human, mouse, budding yeast, frog	yes	yes	no	Scores based on (i) the number of off-targets, (ii) whether off-targets are perfect hits or have mismatches, (iii) location in the gene (the more 5' the better), (iv) GC-content, (v) G at position 20 in the target site.	no
**DNA2.0 gRNA Design Tool** (https://www.dna20.com/eCommerce/startCas9)	–	10 kbp	human, mouse, budding yeast, *E. coli*	yes	no	no	Scores based on the occurrence of the 12 base pair seed sequence in the genome.	no
**Cas-OFFinder** (http://www.rgenome.net/cas-offinder/)	Bae *et al.* (2014) *Bioinformatics*, **30**, 1473-1475.	(performs off-target searches for given gRNAs)	human, mouse, cow, dog, rat, pig, zebrafish, fruit fly, *C. elegans*, *Arabidopsis*, rice, tomato, corn, monkey, *Aspergillus*	yes	yes	no	–	no
**CRISPRdirect** (http://crispr.dbcls.jp/)	**This work**	**10 kbp**	**human, mouse, rat, marmoset, pig, chicken, frog, zebrafish, *Ciona*, fruit fly, silkworm, *C. elegans*, *Arabidopsis*, rice, *Sorghum*, budding yeast**	**yes**	**yes**	**yes**	**Target sites with unique 20mer+PAM and 12mer+PAM in the genome are marked green. A detailed list of off-targets with mismatches and gaps are investigated in separate window.**	**yes**
